# Differences in Peak Knee Flexor Force between Eccentric-Only and Combined Eccentric-Concentric Nordic Hamstring Exercise

**DOI:** 10.3390/sports11020041

**Published:** 2023-02-07

**Authors:** Jesper Augustsson, Håkan Andersson

**Affiliations:** 1 Department of Sport Science, Faculty of Social Sciences, Linnaeus University, 39182 Kalmar, Sweden; 2 High Performance Center, Strength and Conditioning Institute, 352 46 Vaxjo, Sweden

**Keywords:** hamstring, injury, prevention, rehabilitation, return to sport

## Abstract

In many sports, the hamstring strain injury is a common injury. There is evidence that the Nordic hamstring exercise (NHE), a knee flexor exercise, can reduce hamstring injury risk in athletes. In research on hamstring injury prevention, eccentric-only NHE is typically performed, whereas in sports, it is relatively common for athletes to perform NHE eccentrically-concentrically. Further, NHE strength is generally assessed by measuring knee flexor force through an ankle brace, attached atop of a load cell. An alternative method might be to assess knee flexor force about the knee joint using a force plate. The aim of the study was to investigate differences in peak knee flexor force between eccentric-only and combined eccentric-concentric NHE. The purpose was also to determine the correlation between hamstring force measured at the ankle using a load cell (current gold standard) and force assessed about the knee joint using a force plate during NHE. Fifteen junior and senior elite soccer and track and field athletes (3 women and 12 men aged 17–27 years) performed eccentric NHE (ENHE) in which they leaned forward as far as possible until breakpoint and eccentric-concentric NHE (ECNHE) where they returned to the starting position. A linear encoder measured the position at which peak force occurred during the NHEs. Force assessed at the ankle differed significantly (678 vs. 600 N, *p* < 0.05), whereas force about the knee joint did not (640 vs. 607 N, *p* > 0.05) between ENHE and ECNHE (12 and 5% difference, respectively). The forward distance achieved by the participants in cm at breakpoint for ENHE was 37% higher than at the coupling phase for ECNHE (74 vs. 54 cm, *p* < 0.001). Very strong significant (*p* < 0.01) correlations were noted between peak force assessed at the ankle and about the knee joint for ENHE and ECNHE, *r* = 0.96 and *r* = 0.99, respectively. Our results suggest that ECNHE, where peak knee flexor force was reached with 37% less forward movement, may complement ENHE, i.e., during hamstring injury rehabilitation, where a position of great knee extension may not be well tolerated by the athlete. Further, assessing knee flexor force about the knee joint using a force plate may provide an alternative to measuring force at the ankle using a load cell when testing NHE strength.

## 1. Introduction

Hamstring strain injury (HSI) is one of the most common injury diagnoses in many sports, for example, football (soccer) [1] and track and field [2]. According to Kerkhoffs et al. [3], the estimated incidence rate of HSIs per 1000 h of participation was found to be 0.87 in non-contact sports (competitive sprinters), and 0.92–0.96 in contact sports (soccer). HSI reinjury rates are high, ranging from 17 to 34% across soccer [4] and 12 to 43% across football [3]. Further, reinjuries are as a rule more severe than the index HIS [5].

At least two clearly different types of hamstring strains have been described in the literature with different mechanisms of injury [6]. Firstly, hamstring strains that occur during high-speed running, and secondly, stretching-type movements or exercises that lead to a lengthened state of the hamstrings, such as high kicking or slide tackling. Recovery from a stretching-type hamstring injury is reportedly significantly slower than sprinting-type hamstring strains [7].

The most performed exercise in hamstring injury-prevention programs is the Nordic hamstring exercise (NHE) [8]. A recent systematic review and meta-analysis concluded that NHE reduces injuries by 50% in athletes [9], which makes NHE one of most efficient hamstring injury-prevention strategies in all of sports [10]. The principal way the NHE has been executed by athletes in research is by an eccentric-only muscle action (where they gradually lean forward as far as possible until breakpoint and then fall to the floor in a controlled fashion, where they put their hands out to catch themselves). In sports, however, physiotherapists and trainers relatively often have the athletes perform NHE eccentrically-concentrically, in which they lean forward (eccentric phase) and then return to the starting position (concentric phase) [11]. Recently, eccentric NHE (ENHE) training was noted to increase knee flexor fascicle length [12]. While it is still not clear which adaptations are accountable for the preventive effects of NHE training on hamstring injuries, theoretically, longer muscle fascicle length would prevent the muscle from injury because of over-lengthening [13]. However, it is important to note that standard strength training (consisting of a concentric and eccentric phase) also has been noted to increase muscle fascicle length [14,15]. In the hamstring injury-prevention literature, traditional ENHE has yet to be compared to eccentric-concentric NHE (ECNHE) when it comes to, for example, differences in knee flexor force. In maximal efforts, eccentric force has repeatedly been reported significantly higher than concentric in the literature [16,17]. The difference in force between maximal eccentric and eccentric-concentric actions, however, is not well studied in strength-training research [18] and is lacking in studies on NHE. While it could be said that there is an evidence-based preventive effect of NHE training [9], several fundamental issues such as how to perform this exercise when it comes to dosing and whether eccentric-only or eccentric-concentric muscle actions should be used is still not clear.

In the early 2000s when research on ENHE training began to emerge [19], isokinetic dynamometry was commonly used to assess the effect of a ENHE program on hamstring strength [20]. However, the predictive validity of this method for detecting hamstring strain risk has been disputed [21]. More recently, the gold standard test when assessing effects of ENHE training is the NordBord device, originally described by Opar et al. [22]. When an athlete performs an ENHE using the NordBord device, hamstring force is measured by an ankle brace, attached atop of a load cell. To the best of our knowledge, no previous study has instead assessed knee flexor force about the knee joint using a force plate during ENHE (or ECNHE), as an alternative method to the NordBord device. The ability to assess the NHE without the need for a NordBord device may be useful, e.g., for practitioners or laboratories who already have a force plate or plan to invest in one.

The primary aim of the study was therefore to investigate differences in peak knee flexor force between ENHE versus ECNHE. The secondary purpose of this study was to determine the correlation between the hamstring force measured at the ankle using a load cell (current gold standard) and the force assessed about the knee joint using a force plate during both ENHE and ECNHE. We hypothesized firstly that peak knee flexor force would be higher for ENHE when compared with ECNHE, and further, that peak knee flexor force would occur with less amount of forward motion for ECNHE when compared with ENHE. Secondly, it was hypothesized that a strong correlation would exist between peak force measured at the ankle using a load cell and about the knee joint using a force plate for both ENHE and ECNHE.

## 2. Materials and Methods

### 2.1. Trial Design and Experimental Approach

The study had a cross-sectional design in which the testing for a particular participant was completed during a single test session. The participants were evaluated firstly on differences in peak knee flexor force between ENHE versus ECNHE. Secondly, the participants were assessed to determine the correlation between the hamstring force measured at the ankle using a load cell (current gold standard) and about the knee joint using a force plate during ENHE and ECNHE.

**Figure 1:**
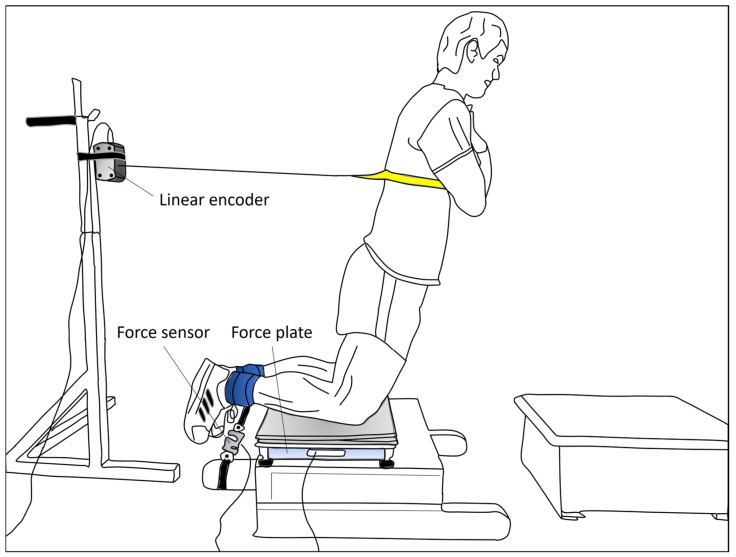
Testing set-up. The participants were positioned onto the custom-made Nordic hamstring exercise (NHE) device, on their knees over the padded board, with their ankles secured by straps and arms crossed over their chest. A linear encoder measured the position at which position peak force occurred during maximal trials of eccentric-only and combined eccentric-concentric NHE, respectively. Hamstring force was measured at the ankle using a load cell and the force assessed about the knee joint using a force plate during the two different NHEs.

ENHE and ECNHE force were measured by using a custom NHE device specifically designed for this study (see Figure 1). Hamstring force was measured at the ankle using a load cell (MuscleLab, Ergotest Technology AS, Langesund, Norway) and the force assessed about the knee joint using a force plate (MuscleLab, Ergotest Technology AS, Langesund, Norway) during ENHE and ECNHE. A linear encoder (MuscleLab, Ergotest Technology AS, Langesund, Norway) attached to the participants’ torsos documented the position at which position peak force occurred during ENHE and ECNHE, respectively.

### 2.2. Participants

Fifteen elite athletes (competing at both national and international levels) consisting of youth male soccer players (*n* = 10) and female (*n* = 3) and male (*n* = 2) track and field athletes, aged 17–27 years, participated in this study (see Table 1 for the characteristics of participants). The study was carried out during the participants’ non-competitive, off-season period. The inclusion criteria included being a well-trained athlete experienced with the NHE in regular training. The exclusion criteria included knee, hip, or back injury before testing within 6 months of the study. Prior to inclusion, all athletes were informed of the risks and benefits of participation before providing written informed consent.

### 2.3. Procedures

NHE testing. The participants performed a general warm-up that consisted of 5 min of body-weight squats, standing calf raises, and hip raises. The participants were then placed onto the NHE device in a kneeling position over the padded board, with their ankles secured with ankle straps and arms across their chest. The padding consisted of a layer of two 1,1 cm thick foam pads, chosen to prevent any discomfort for the participants during testing and yet thin enough not to affect force distribution about the knee to the force plate. Three submaximal ECNHE repetitions (approximately at 50% effort) in which participants were instructed to gradually lean forward in a slow, controlled manner (eccentric phase) and then return to the starting position (concentric phase) were then performed. Three submaximal, eccentric phase only, ENHE repetitions (approximately at 80% effort) followed where the subjects leaned forward, and then, in a controlled manner, fell to the floor and put their hands out to catch themselves. A linear encoder (MuscleLab, Ergotest Technology AS, Langesund, Norway), fixated to a squat rack placed behind the participants at a height of 117 cm, was then connected to the subjects’ torsos through a strap, at a height standardized to 80 cm above the knees. The linear encoder measured the position at which peak force occurred during ENHE and ECNHE, respectively. Subsequently, two to three maximum trials of ENHE and ECNHE repetitions, respectively, were performed, separated by 1 min of rest. Hamstring force was measured simultaneously at the ankle using a load cell (MuscleLab, Ergotest Technology AS, Langesund, Norway) and the force assessed about the knee joint using a force plate (MuscleLab, Ergotest Technology AS, Langesund, Norway) during ENHE and ECNHE. The force sensor was placed between the ankle straps and the NHE device. The sample rate was 200 Hz, and further, no filter was used as an analog-to-digital converter for each signal for the linear encoder, load cell, and force plate. The test order was randomized across the participants, so that half the participants performed ENHE first and ECNHE last, and vice versa, using the Microsoft Excel RAND function to generate evenly distributed random numbers. Verbal encouragement cues and commands to the participants were standardized. One of the investigators, a physical therapist who had over 25 years of experience of strength training and testing, supervised all tests and the performance of all trials. Each trial was only considered as successful if the subjects held the trunk and hips in a neutral position throughout the NHE repetitions. Data from the linear encoder was synchronised with the load cell and the force plate through the MuscleLab system (V10.21, Ergotest Technology AS, Langesund, Norway).

### 2.4. Statistical Analyses

Data were analyzed using IBM SPSS Statistics (version 26, IBM, Armonk, NY, USA). A Shapiro–Wilk test was conducted and revealed that the considered data was normally distributed and could be analyzed with parametric tests for significance (*p* > 0.05). The results are presented as mean with SD. Paired samples *t*-tests were used to detect significant differences in peak force assessed at the ankle and about the knee joint between ENHE and ECNHE, respectively. Analyses of differences in the forward distance achieved by the participants in cm at breakpoint for ENHE versus at the coupling phase, i.e., the phase between the eccentric and concentric phases [23], for ECNHE was performed using the paired samples *t*-tests. The Cohen *d* effect size (ES) was calculated to indicate the difference in peak knee flexor force, assessed at the ankle and about the knee joint, and forward distance achieved by the participants between ENHE and ECNHE using the following formula: mean ENHE—mean ECNHE divided by the pooled SDs of the two variations of the exercise. An ES of 0.2 was considered small, 0.5 represented a medium ES, and 0.8 a large ES [24]. To examine the relation between peak force assessed at the ankle and about the knee joint for ENHE and ECNHE, respectively, Pearson product-moment correlation coefficients were determined. The strength of the correlations was evaluated using the following categorization: *r* = 0.00–0.10, insignificant correlation; *r* = 0.10–0.39, weak correlation; *r* = 0.40–0.69, moderate correlation; *r* = 0.70–0.89, strong correlation; and *r* = 0.90–1.00 very strong correlation [25]. Sample size calculation: The number of participants in the study was determined based on a hypothesized 15% difference in peak knee flexor force between ENHE and ECNHE. The estimated minimum number of participants was 15, with a statistical power of 0.80. The significance levels for all analyses were set to *p* < 0.05.

## 3. Results

**Figure 2:**
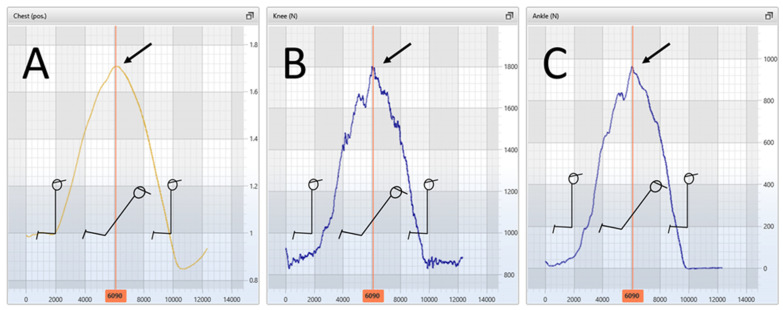
Representative illustration of a participant in which, for the eccentric-concentric Nordic hamstring exercise (NHE), arrows indicate that peak knee flexor force assessed at the ankle (panel (**C**)) and about the knee joint (panel (**B**)) occurred at/matched the maximal forward distance achieved, i.e., during the coupling phase (the phase between the eccentric and concentric phases) (panel (**A**)).

Force assessed at the ankle differed significantly (678 vs. 600 N, *p* < 0.05, ES = 0.56), whereas force about the knee joint did not (640 vs. 607 N, *p* > 0.05, ES = 0.24) between ENHE and ECNHE (12 and 5% difference, respectively). The forward distance achieved by the participants in cm at breakpoint for ENHE was 37% higher than at the coupling phase for ECNHE (74 vs. 54 cm, *p* < 0.001, ES = 1.9). The peak force values and the forward distance achieved by the participants for the two different types of NHE are displayed in Table 2. For the ECNHE, peak knee flexor force occurred at/matched the maximal forward distance achieved, i.e., the coupling phase, for every participant (see Figure 2).

**Figure 3:**
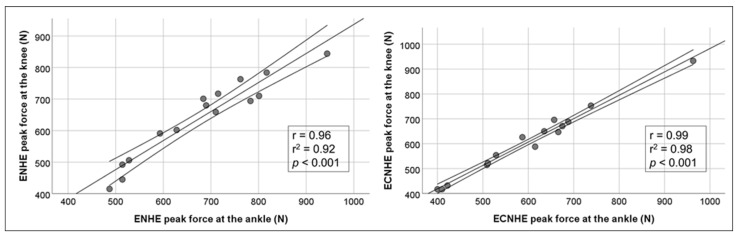
Correlations between peak force assessed at the ankle and about the knee joint for eccentric-only and combined eccentric-concentric Nordic hamstring exercise (NHE), respectively, in junior and senior elite athletes (*n* = 15) with 95% confidence interval.

Very strong significant (*p* < 0.01) correlations were noted between peak force assessed at the ankle and about the knee joint for ENHE and ECNHE, *r* = 0.96 and *r* = 0.99, respectively. Figure 3 illustrates the correlations between peak force assessed at the ankle and about the knee joint for ENHE and ECNHE, respectively.

## 4. Discussion

The main findings of this study were that peak force differences between ENHE and ECNHE were relatively small (12 and 5% difference, respectively), and that for ECNHE, peak knee flexor force was reached with 37% less range of movement. Further, very strong significant (*p* < 0.01) correlations were noted between peak force assessed at the ankle and about the knee joint for ENHE and ECNHE, *r* = 0.96 and *r* = 0.99, respectively.

This is, to our knowledge, the first study investigating the effect of NHE mode, i.e., eccentrically-only, or eccentrically-concentrically, on peak force and amount of forward motion during these two variations of the exercise. Although the forward distance achieved by the participants in cm at breakpoint for ENHE was significantly higher than at the coupling phase for ECNHE (37%, *p* < 0.001), the difference in peak force was relatively small (12% when measured at the ankle and 5% difference when measured about the knee joint). Thus, ECNHE may reach force values that are comparable to traditional ENHE with significantly less range of movement. Because ECNHE involves movement at shorter hamstrings length, it may be more well tolerated by, for example, an athlete during hamstring injury rehabilitation and a less strenuous alternative for a recreational athlete. Further, compliance with ENHE hamstring injury prevention programs has reportedly been low [26]. One reason for the low compliance may be that ENHE causes muscle soreness in athletes and, therefore, in the short term impedes, e.g., sprint performance [27]. Further studies that investigate to what extent ECNHE causes muscle soreness in athletes are therefore desirable.

The most common method for assessing knee flexor strength after anterior cruciate ligament (ACL) reconstruction is by isokinetic dynamometry, performed concentrically while seated [13]. In recent years, however, ENHE has been used to test limb-to-limb differences in muscle strength to assist in determining return to sport in patients after ACL reconstruction [13,28]. It may be that ECNHE training and testing at short hamstring lengths could act as a less strenuous alternative during the early and mid-phases of ACL rehabilitation to ENHE training and testing at long hamstring lengths.

Whilst we recognize the importance of the NordBord device, the current gold standard to determine the outcome of NHE training in practice and in research, it is fairly high in cost. We show in our study that a force plate (a device that has multiple uses in sports and research) may serve the function of measuring NHE strength as well. From a practical standpoint, the ability to assess the NHE without the NordBord device is useful, e.g., for researchers, strength professionals, practitioners, or laboratories who already have a force plate or plan to invest in one.

Exercises can be categorized as either static or dynamic and involve muscle actions that are isometric, concentric, and/or eccentric. [16]. Tests of maximal strength in research and sports, exercises such as the bench press and the barbell squat for example, are as a rule evaluated eccentrically-concentrically. An inability to either concentrically press the bar from the chest to straight arms (bench press) or stand erect with extended knees at the end of the movement (barbell squat), is cause for failure of the respective lifts. In fact, it is so common to perform the concentric part of strength exercises that the activities themselves have names that imply the involvement of concentric movement: weight*lifting*, power*lifting*. The exception to this rule would be NHE research in which, as mentioned earlier, the typical NHE variation is for the athletes to perform the exercise eccentrically [8,12,20,22,29,30,31]. However, this differs from the usual practice in strength training and testing, where key exercises like the bench press and barbell squat generally are performed eccentrically-concentrically. Advantages of using both eccentric and concentric muscle actions during NHE testing and training as opposed to just eccentric ones could include increased workload (performing a “full” repetition rather than a “half” repetition), and a closer representation of athletic movements like running and jumping, as the hamstrings are engaged both eccentrically and concentrically during ECNHE.

The peak force differences between ENHE and ECNHE were relatively small, even though peak knee flexor force was reached with 37% less range of movement for the ECNHE. If we analyze this result, there is at least one explanation for how this was possible mechanically. For the ECNHE, peak knee flexor force occurred at/matched the maximal forward distance achieved for every participant, i.e., at the coupling phase (see Figure 2). It may be that while the eccentric-deceleration phase of ECNHE was not performed in a plyometric, explosive manner by the participants, the hamstring muscle was nevertheless preloaded eccentrically [32]. This may have resulted in fairly large amounts of momentum and force being produced at the coupling phase and the concentric-acceleration muscle action that followed, force that was relatively close to the magnitude of ENHE force. In other words, even though there was no stretch-shortening cycle type of muscle action, like jumping or hopping, the ECNHE muscle action was rather large in force.

The current study has some limitations. To reduce the risk of injury, the participants performed a warm-up that included performing submaximal repetitions of NHE prior to testing. However, we believe the fatiguing effect of the warm-up on NHE test performance to be negligible. Also, the reliability of the tests of NHE strength was not calculated. In a previous study on female soccer players, however, we evaluated test-retest reliability using the same NHE test-setup and found excellent reliability (the intra-class correlation coefficient was 0.95) [11]. Further, very strong significant (*p* < 0.01) correlations were noted between hamstring force measured at the ankle using a load cell and force assessed about the knee joint using a force plate during NHE. We do not, however, recommend using the two methods—load cell and force plate—interchangeably when testing NHE strength in athletes but rather adhere to one of them. Lastly, the participants could be considered somewhat heterogeneous as they consisted of both women and men, ranging from 17 to 27 years old and were either soccer players or track and field athletes. It is important to note, however, that they did not differ when it comes to the inclusion criteria: being a well-trained athlete, experienced with the NHE in regular training.

When it comes to further research on NHE, we suggest that a key research path is investigations on a stepwise NHE progression (that would include exercise variations such as ECNHE and ENHE) that might allow athletes with hamstring injuries to return to play more safely and effectively. Additionally, although the preventive effect of NHE on hamstring injuries has been researched over 20 years [19], there is still no consensus on fundamental training variables such as, e.g., optimal dosing. NHE interventions on athletes in which training volume and NHE variations depended on the season would be interesting to study. For instance, by investigating a concept of programming that entailed higher training volume and more focus on ENHE during off-season and lower training volume and more accentuation on ECNHE during the competitive season.

## 5. Conclusions

Our results suggest that ECNHE, where peak knee flexor force was reached with 37% less forward movement, may complement ENHE, i.e., during hamstring injury rehabilitation, where a position of great knee extension may not be well tolerated by the athlete. This is essential knowledge, e.g., for a sports physical therapist, and may benefit an athlete in their hamstring rehabilitation progression.

Further, assessing knee flexor force about the knee joint using a force plate may provide an alternate to measuring at the ankle with a force cell when testing NHE strength. From a practical point of view, a force plate can therefore be recommended as an alternative method to assess NHE knee flexor strength in athletes. The ability to test NHE strength with a force plate is valuable, e.g., for researchers, strength professionals, practitioners, or laboratories who already have a force plate or plan to invest in one.

## Figures and Tables

**Table 1:** Characteristics of participants (*n* = 15).

Characteristics	*n*	Mean ± SD
Female	3	
Male	12	
Soccer players	10	
Track and field athletes	5	
Age, year		19 ± 3
Height, cm		182 ± 8
Weight, kg		74 ± 10
Practice, hours per week		11 ± 2

**Table 2:** Peak force values and forward distance (mean ± SD) achieved by the participants for eccentric-only versus combined eccentric-concentric Nordic hamstring exercise (NHE).

Test Difference	Eccentric-Only NHE	Combined Eccentric-Concentric NHE
Peak force at the ankle (N)	678 ± 133 *	600 ± 147
Peak force about the knee joint (N)	640 ± 128	607 ± 139
Forward distance achieved (cm)	74 ± 10 *	54 ± 11

* Different from combined eccentric-concentric NHE, *p* < 0.05.

## Data Availability

The data presented in this study are available on request from the corresponding author.
